# Obstructive uropathy secondary to ureteral inguinoscrotal hernia

**DOI:** 10.1590/S1677-5538.IBJU.2019.0199

**Published:** 2020-01-13

**Authors:** Isabel Senra Bravo, Luis García Martín, Pablo Garrido-Abad, Almudena Coloma del Peso, Diego Enjuto Tristán, Manuel Fernández Arjona

**Affiliations:** 1 Department of Urology, Hospital Universitario Del Henares, Coslada, Universidad Francisco de Vitoria, Madrid, Spain;; 2 Department of Surgery, Hospital Universitario Del Henares, Coslada, Madrid, Spain

## CASE PRESENTATION

An 83-year-old male patient with a medical history of benign prostate hyperplasia was admitted with left inguinoscrotal pain and swelling. Physical exam revealed a large left-sided irreducible inguinoscrotal hernia (ISH). A non-contrast abdominopelvic computer tomography showed a left hydroureteronephrosis with a dilated ureter included in a paraperitoneal ISH associated with left nephroptosis. Renal function was normal, with a creatinine level of 0.92mg/dl.

Subsequent surgical repair by hernioplasty with a synthetic mesh placement was performed in a multidisciplinary approach. Intraoperatively, we found a large paraperitoneal ureteral ISH with dilated gonadal vessels. Patient was discharged after 3 days without complications. The patient is free of symptoms after 3 months of follow-up and the IVU showed hydroureteronephrosis resolution.

## DISCUSSION

Inguinoscrotal herniation (ISH) of the ureter is extremely rare, even more on native kidneys (1). ISH is common in 50-60 years old men and frequently associated with pelvic kidney (2). We can find both paraperitoneal (80%) and extraperitoneal presentation (20%) (3). Paraperitoneal type has a peritoneal indirect sac that pulls the ureter through the defect, forming part of the hernia wall, due to a layer of posterior peritoneum. Extraperitoneal type is characterized by containing no peritoneal sac and the ureter is involved alone or with retroperitoneal fat (3).

**Figure 1:**
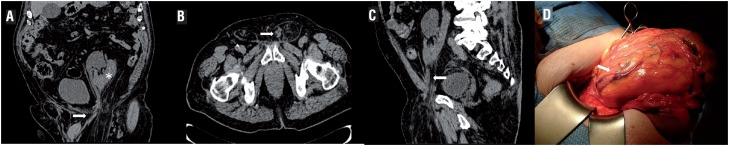
A) Coronal abdominal non-contrast-enhanced CT scan showing dilated left pelvic kidney (asterisk) and dilated ureter (arrow) in the inguinal hernia. B) Axial non-contrast-enhanced CT scan with loop of the left ureter in the hernia (arrow). C) Sagittal non-contrast-enhanced CT scan with ureteral inguinoscrotal hernia (arrow). D) Identification of left ureter (arrow) during the inguinal hernia repair.

**Figure 2:**
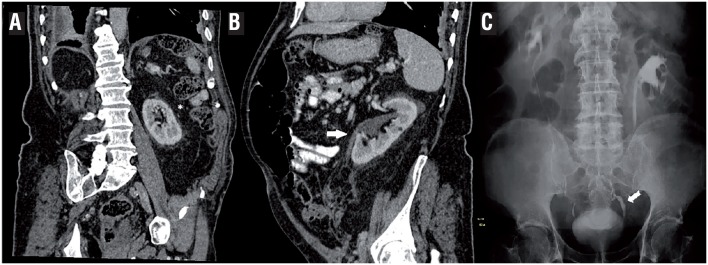
A) Coronal-oblique post-contrast-enhanced abdominal CT scan showing left pelvic kidney (asterisk) without dilated ureter after correction of the inguinal hernia. B) Sagittal CT scan showing non dilated left pelvic kidney (arrow). C) Intravenous pyelogram post hernioplasty.

This condition usually has an asymptomatic course unless ureteral obstruction causes pain, infections or renal dysfunction (1, 3), signs that indicate ISH should be considered. Computed tomography scan helps to delineate the course of the ureter (3). Treatment modalities consist of surgical repair (1–3).
